# Analysis of Hydration Habits Before and During a Specific Training Session in Male Padel Athletes Aged over 65: Physiological and Psychological Implications

**DOI:** 10.3390/nu16203513

**Published:** 2024-10-16

**Authors:** Ana Júlia Lopes, Maria João Campos, Fátima Rosado, Luís Rama, Alex Silva Ribeiro, Diogo Martinho, Ana Teixeira, Alain Massart

**Affiliations:** 1 Faculty of Sport Sciences and Physical Education, University of Coimbra, 3040-248 Coimbra, Portugal; ho_quei_9@hotmail.com (A.J.L.); mrosado@fcdef.uc.pt (F.R.); 2 Research Unit for Sport and Physical Activity (CIDAF), Faculty of Sport Sciences and Physical Education, University of Coimbra, 3040-248 Coimbra, Portugal; luisrama@fcdef.uc.pt (L.R.); alexribeiro@fdef.uc.pt (A.S.R.); diogomartinho@fdef.uc.pt (D.M.); ateixeira@fcdef.uc.pt (A.T.); alainmassart@fcdef.uc.pt (A.M.)

**Keywords:** aging, exercise, nutrition, health

## Abstract

(1) Background: Since older adults are more susceptible to dehydration and there is a lack of information on older athletes, this study observed a group of 12 male padel players in this age group (70.42 ± 3.50 years) to characterize their hydration habits, physiological demands, and psychological responses before and during a 90 min padel training (PT). (2) Methods: After approval from the Ethics Committee (CE/FCDEF-UC/00022023) and the provision of signed informed consent, participants’ body mass, height, waist and hip circumferences, body mass index, waist-to-hip ratio, and waist-to-height ratio were measured. Habitual fluid intake was monitored by diary from the evening until before the PT; the subjects completed a Profile of Mood States questionnaire (POMS) and a satiety scale (SLIM). To assess hydration levels at different moments, we used a portable osmometer and an eight-point urine color chart and weighed the participants immediately before and after the PT. During the PT, heart rate (HR) and hydration were monitored. After the PT, subjects completed another POMS and SLIM. (3) Results: Subjects trained at 73.2 ± 12.3% of their maximum HR, with brief peaks at the anaerobic threshold or higher (130.00 ± 18.78 bpm). The mean urine osmolality indicated normal hydration or minimal dehydration. However, the urine color values indicated dehydration after the training. Subjects drank 438 mL of liquids at night, 333 mL before PT, and 900 mL during the PT, with a good repartition of the liquids. POMS and SLIM were not affected by the training. (4) Conclusions: Older male padel athletes achieved challenging yet safe training, staying within healthy intensity zones; their hydration patterns nearly met the recommendations for exercise and should be slightly increased.

## 1. Introduction

Padel has experienced exponential growth in popularity over the last decade, becoming a sport practiced worldwide. Recent studies suggest that padel can be an effective activity for promoting health and improving physical activity during leisure time in sedentary populations [1,2].

This sport intermittently uses sprints, agility, and changes in speed, leading to differences in physiological responses (lactate, heart rate, perceived effort) during play [1,3,4]. Depending on their skill level, padel athletes exhibited an average heart rate (HR) ranging from 68% to 85% of their maximum HR, with peak heart rates during matches between 154 and 179 bpm. Lactate levels were measured between 2.40 and 3.38 mmol/L, and Borg scale ratings ranged from 3.84 to 5.85, indicating a predominantly aerobic effort.

For athletes of any age, negative effects on athletic performance have been demonstrated even with modest dehydration (2%) [5]. With dehydration, the sweat rate decreases, and thermoregulation and cardiovascular function are impaired, leading to increased heart rate, subjective perception of effort, and core body temperature [6]. Thermoregulation capabilities diminish with age, making older adults more susceptible to dehydration, which can result from insufficient water intake or excessive water loss [7,8,9]. In older athletes, susceptibility to dehydration and its subsequent negative effects on thermoregulation and athletic performance may be increased due to numerous age-related changes, such as disturbances in thirst mechanisms, renal function, and sweating, which can inhibit the ability to consume adequate fluids and cope with the physiological stresses of exercise in older practitioners [10,11]. This implies that older adult athletes should be educated about timely and adequate fluid intake before, during, and after physical exercise [12,13].

Studies on nutrition in padel are scarce and have only been related to supplementation [14]; there is a lack of research on hydration adequacy and its impact on the functional capacities of older adults during physical exercise [12,15]. We did not find any studies on the hydration of older padel players during exertion or on their physiological demands during this sport.

The aim of this study is to observe the hydration habits of a group of trained senior men before and during padel training and to relate their fluid consumption to the physiological and psychological demands of the training. We hope to contribute to a better understanding of hydration recommendations for exercise in older individuals, particularly in padel, as this is the first study on this topic.

By examining the hydration strategies of older padel athletes, this study aims to contribute to the growing body of literature focused on optimizing athletic performance and health in an aging population. Understanding the interplay between hydration and the specific demands of padel will provide valuable insights for coaches, trainers, and athletes alike.

This study addresses the critical issue of dehydration susceptibility in older athletes, an area that has received limited attention in sports science. By specifically examining older padel players, our research sheds light on the nutritional needs of aging athletes, particularly in maintaining hydration and preventing performance decline, which are key themes within the journal’s scope.

## 2. Materials and Methods

### 2.1. Participants

The study sample consisted of veteran male padel players over the age of 65 who are healthy, regular practitioners and members of the Portuguese Padel Federation. Given the specific characteristics of this group and their relatively small number, they train at various clubs in the same city and have taken the initiative to train together on weekends. According to the information gathered, the group with these specific characteristics consists of no more than 15 people, only males, of whom 12 agreed to participate in this study, which prevented the use of a control group and limited this study to a case study with a convenience sample.

### 2.2. Procedures

After approval from the Ethics Committee (CE/FCDEF-UC/00022023), the participants were recruited through a representative of the veteran athletes’ group. Following a meeting with the volunteers, during which all the study procedures were explained in detail, each athlete was given an informed consent document, which was returned signed by all study participants one week later. Subsequently, to prepare the voluntary athletes for the experimental phase of this study, they were measured (body mass, height, and circumferences), questioned (regarding age, medication intake, and weekly hours of physical exercise), and introduced to the study procedures (completion of questionnaires, urine collection, use of heart rate monitors, and clarifications about the sequence of tasks on the experimental day). This study is in line with the ethical aspects of the Declarations of Helsinki 2013.

### 2.3. Study Protocol

Participants arrived at 9:30 am, and the urine containers from the previous day and the morning, along with hydration diaries, were collected. They then completed the Profile of Mood State questionnaire (POMS pre-training) and the satiety scale (pre-training). This was followed by the collection of pre-training urine, body mass measurements, and the fitting of heart rate monitors. After these procedures, participants followed their usual training routine. During the session, they were instructed to drink water “ad libitum” according to their normal habits. Each player was provided with a 1.5 L individual water bottle. At three points during the session, the assessors recorded the amounts of water consumed to gauge the drinking pace and total volume consumed by each athlete during training. After the session, post-training urine was collected and body mass was measured. This was followed by the completion of the POMS questionnaire (post-training) and the satiety scale based on feelings during the session. Throughout the experimental phase, a club doctor was present. Urine collection was conducted in a private setting, and the questionnaires were completed individually without interference from training partners. All tools used during the protocol identified participants by a randomly assigned code.

The training session consisted of 15 min of warm-up carried out as a group, covering the various essential aspects of the sport: joint mobility and the strokes used during training/matches, forehand/backhand strokes, forehand/backhand glass (wall), forehand/backhand volleys, tray, and smash. This was followed by 75 min of practice matches, played in 3 sets with a rest interval.

### 2.4. Measures

#### 2.4.1. Anthropometric Measurements

Body mass was monitored using a portable digital scale (SECA 878, SECA, Hamburg, Germany) immediately before and after the training session, with participants barefoot and wearing a dry t-shirt. The subjects’ height was measured one week before the experimental day, during the preparation for the study procedures, using a portable stadiometer (Bodymeter 206 SECA). On the same day, waist and hip circumferences were measured using a plastic measuring tape for perimeters (SECA model 201, 2 m). The anthropometric procedures described by [16] were followed in this study. Based on these measurements, body mass index (BMI) (kg/m²), waist-to-hip ratio (cm/cm), and waist-to-height ratio (cm/cm) were calculated. In terms of health risk, in older male adults, the following values could be considered as clinical normal risk: body mass index of 27–28 kg/m² [17], waist circumference of 92–106 cm [18], waist-to-hip ratio of ≤ 0.95 [19], and waist-to-height ratio of 0.47 to 0.56 [20].

#### 2.4.2. Heart Rate Monitoring

Before the training session, each participant was fitted with a heart rate monitor with a heart rate sensor (Polar RS800CX and Polar V800 with GPS, Polar, Kempele, Finland) to record heart rate, distances covered, and running speeds throughout the entire session. The percentage of maximal heart rate was assessed using the age-predicted maximal heart rate, calculated with the equation HRmax = 220 − Age, as recommended for the general population [21].

#### 2.4.3. Hydration Level Monitoring

As in other studies on athletes [22,23], the collected urine was analyzed using a portable osmometer (Osmocheck VITECH Scientific Ltd., Horsham, UK), which is a refractometer calibrated in mOsmols/kg H_2_O to measure urine osmolality from 0 to 1500 mOsmols/kg H_2_O, with an accuracy of 10 mOsmols/kg H_2_O. The urine analysis with the Osmocheck was conducted according to the manufacturer’s recommendations (VITECH Scientific Ltd., Billingshurst, UK). After it was ensured that the Osmocheck device was calibrated, clean, dry, and ready for use, a urine sample was collected in a sterile container. Small quantities of the sample (20 µL) were extracted with a pipette and transferred into the sample well of the osmometer, ensuring no air bubbles or spillage. Measurements were repeated at least twice for accuracy. To evaluate hydration levels, the American College of Sports Medicine recommends values of ≤700 mOsm/kg to ensure hydration [24]. Due to variability found in studies, other authors suggest a normal hydration range of 600 to 820 mOsm/kg, with warning levels between 820 and 1000 and danger levels above 1000 mOsm/kg [25,26].

Urine color was determined via the eight-point urine color chart developed by Armstrong, values < 3 can be considered representative of normal hydration; 4–6, dehydration; 7–8, severe dehydration [27]. Despite some limits, this method continues to be an easy-use alternative [28,29].

Weighing the participants immediately before and after the training session allowed for a supplementary assessment of hydration levels during the session, as recommended in athletes [30].

#### 2.4.4. Assessment of Fluid Intake

From the last dinner on the evening before until the start of the training session, participants were asked to record all liquid intake in a hydration diary, using household measurements adapted for the Portuguese population [31]. During the training, the participants’ “ad libitum” hydration was monitored at three points by the research team, evaluating the remaining water in the bottles provided to the individuals.

#### 2.4.5. Administration of POMS Questionnaire and SLIM Scale

The score of the POMS questionnaire, that is, the “Profile of Mood States” questionnaire, often used to assess mood, is calculated by summing the totals for the negative subscales (tension, depression, fatigue, confusion, anger) and then subtracting the totals for the positive subscales (vigor). The Satiety Labeled Intensity Magnitude (SLIM) scale describes different levels of fullness from 0 to 100 and hunger from 0 to minus 100, along a vertical line. The Portuguese version of the POMS questionnaire [32] was administered immediately before and after the training session to measure the athletes’ mood and fatigue levels throughout the session. The SLIM satiety scale by Cardello, translated and adapted into Portuguese [33], was used immediately before and after the session to better assess the athletes’ comfort regarding the drinks consumed before and during training.

### 2.5. Statistical Analysis

The statistical analysis was conducted using IBM SPSS Statistics 26. We used mean, standard deviation, and frequency (%) in the descriptive part. Initially, we performed the Shapiro–Wilk normality test, which indicated that the distribution of our data did not permit the use of parametric tests. Therefore, we employed non-parametric statistics, based on a significance level of *p* ≤ 0.05. The non-parametric Friedman test was used, with pairwise comparisons adjusted using Bonferroni correction, to compare the results from the different assessment time points. The Wilcoxon test was used to compare results from the same subjects at two time points, and Spearman’s Rank Correlation Coefficient was used to assess relationships between the study variables. Covariance analysis was performed to control for confounding factors, ensuring a more accurate interpretation of these relationships. When we obtained significant results, we calculated the effect size by dividing the z value by the square root of the number of observations (*n*) or by dividing the qui square (χ^2^) value by the number of observations (*n*) multiplied by the number of conditions (k), considering Cohen’s values for small, medium (0.3–0.50), or large effects [34].

## 3. Results

### 3.1. Sample Characterization

The study sample consisted of 12 trained older male subjects, engaging in 4.33 ± 2.50 h of weekly exercise, with a mean age of 70.42 ± 3.50 years, an average body mass of 81.79 ± 7.44 kg, and a height of 1.73 ± 0.03 m. The mean body mass index (BMI) was 27.28 ± 2.14, with a waist circumference of 100.88 ± 6.42 cm, a waist-to-hip ratio of 0.99 ± 0.05, and a waist-to-height ratio of 58.29 ± 3.66. Of the sample, 75% were on medication, with 58.3% taking medication for blood pressure, 25% for cholesterol, and 16% for diabetes.

### 3.2. Characterization of Padel Training Effort

The training session lasted 90 min; according to Table 1, the average effort was 73.17 ± 12.31% of maximum heart rate (HRmax). The average absolute heart rate was 107.00 ± 19.11 bpm, with peak values reaching 130.00 ± 18.78 bpm, representing 88.50 ± 11.69% of HRmax. The average heart rate recovery over one minute during the game was 13.25 ± 8.70 bpm. Using the heart rate training zone [35,36], on average, subjects spent 16.3% of the training time in very light exertion, 29.2% in light exertion, 30.3% in moderate exertion, 15% in high exertion, and 5.5% in maximum exertion. During the training session, the subjects covered an average distance of 1.95 ± 0.37 km and reached maximum speeds of 10.51 ± 1.25 km/h. The recorded temperature and humidity during the session ranged between 17° and 21° and between 61% and 83%, respectively.

### 3.3. Hydration Levels

According to Table 2, the urine osmolalities from the night before, in the morning, pre-training, and post-training had means of 797.50, 778.33, 772.50, and 800.00 mOsm·kg^−1^, respectively, showing no statistically significant differences in the Friedman test: Chi-square = 1.103, *p* = 0.776 (ns).

The mean urine colors show statistically significant variations between the time points (Friedman test: Chi-square = 13.286, *p* = 0.004, small size effect). Pairwise comparisons adjusted using Bonferroni correction reveal that the differences are significant between pre-training and post-training urine colors (*p* = 0.011), between morning and post-training urine colors (*p* = 0.018), and between the night before and post-training urine colors (*p* = 0.003).

Body mass decreased significantly during training (Wilcoxon test: Z = −2.405; *p* = 0.016, large size effect).

The correlations between urine osmolalities and urine color were as follows: at night (*r* = 0.372, *p* = 0.234 ns, with a covariance of 90, and moderate effect), in the morning (*r* = 0.618, *p* = 0.032, with a covariance of 132.57, and large effect), pre-training (*r* = 0.540, *p* = 0.07 ns, with a covariance of 89.77, and large effect), and post-training (*r* = 0.481, *p* = 0.113 ns, with a covariance of 173.64, and moderate effect).

### 3.4. Liquid Intake

The results in Table 3 indicate that the subjects consumed an average of 438 mL of liquids at night (from dinner until sleeping), 333 mL from waking up until breakfast, and 900 mL during the training. The amounts of water ingested at 30, 60, and 90 min of training did not show statistically significant differences (Friedman test: Chi-square = 1.850, *p* = 0.397).

The correlation between the amount of liquid consumed at night and the urine osmolality (Spearman test: *r* = −0.136, *p* = 0.674 ns, with a covariance of −6.08, and weak effect) and that between liquid intake from waking until breakfast and pre-training urine osmolality (Spearman test: *r* = −0.389, *p* = 0.211 ns, with a covariance of −32.05, and moderate effect) both showed negative relationships, though neither was statistically significant. Additionally, the correlation between the change in urine osmolality (post-training minus pre-training values) and total water consumption during training (TWT) was negative and approached statistical significance (Spearman test: *r* = −0.555, *p* = 0.061 ns, with a covariance of −10.91, and large effect). TWT showed a significant negative correlation with the percentage of training time spent in zone 2 (Spearman test: *r* = −0.664, *p* = 0.040, with a covariance of −4.23, and large effect). Although not statistically significant, the correlation and covariance between TWT and the percentage of training time in zones 1 and 3 was also negative, while it was positive in zones 4 and 5. We also examined the correlations and covariance between training hours per week and heart rate, hydration, and POMS results but did not find any significant relationships. However, we did observe a borderline negative correlation between training hours per week and both absolute maximal heart rate (Spearman test: *r* = −0.537, *p* = 0.072 ns, with a covariance of −26.91, and large effect) and the time spent in high-exertion heart rate zones (Spearman test: *r* = −0.623, *p* = 0.054 ns, with a covariance of −24.33, and large effect).

### 3.5. State of Satiety

There was no statistically significant difference (Wilcoxon test, *p*= 0.136, Z = −1.490, moderate size effect) in the state of satiety among the subjects between before training and immediately after training (see Table 4).

### 3.6. Profile of Mood States

The subjects’ mood profiles did not show any overall disturbance due to the effect of training (Wilcoxon test: Z = −0.118, *p* = 0.906). However, under the effect of training, the subjects exhibited a statistically significant decrease in hostility (Wilcoxon test: Z = −2.032, *p* = 0.042, large size effect) and a statistically significant increase in fatigue (Wilcoxon test: Z = −2.255, *p* = 0.024, large size effect). (see Table 5). Although not statistically significant, the correlation and covariance between the evolution of fatigue (post-training minus pre-training fatigue level) and the percentage of time training in zones 4 and 5 was positive (*r* = 0.437 with covariance of 29.99 and *r* = 0.390 with covariance of 9.28, medium size effect, respectively).

## 4. Discussion

A review of the scientific literature (PubMed, Google Scholar) suggests that the present study is likely the first to analyze senior padel players in terms of their training and hydration habits.

### 4.1. Subjects and Training Characteristics

The study sample consisted of trained male seniors with an average age of 73.17 ± 12.31 years. In terms of health risk, the study participants had an average body mass index (27.28 ± 2.4 kg/m²), which corresponds to an overweight status for the general population [35] but could be considered normal in older adults [17]. The average waist circumference was 100.88 ± 6.42 cm, which could be considered a risk or lower risk factor for the general population [35,36] and a normal risk value for older adults [18]. The average waist-to-hip ratio of 0.99 ± 0.05 and the waist-to-height ratio of 58.29 ± 3.66 could both indicate a health risk in our subjects [19,20].

Considering they trained an average of 4.33 ± 2.50 h per week and were able to endure a 1 h 30 min training session without showing heart rate levels of concern, with an in-training average heart recovery rate of 13.25 ± 8.7 bpm per minute, consistent with good health [37], we can conclude that the players in our study exhibited normal health for their age.

During the training, participants exerted an average of 73.2 ± 12.3% of their maximum heart rate (107.00 ± 19.11 bpm), representing a predominantly moderate aerobic effort. There were brief peaks at the anaerobic threshold (15% of the training time) and at maximum aerobic effort (5.5% of the training time), with heart rates averaging no more than 88.5 ± 11.7% of their maximum (130.00 ± 18.78 bpm). This set of exertions led to a statistically significant increase (Wilcoxon test: Z = −2.255, *p* = 0.024) in medium fatigue subscale (POMS) values, from 3.42 ± 4.76 to 6.67 ± 4.62 between the start and end of the session. These data suggest that the training, despite being of moderate intensity, had a significant impact on the players. This is confirmed by the statistically significant decrease in body mass (390 g) between pre- and post-training (Wilcoxon test: Z = −2.405, *p* = 0.016), despite substantial water consumption (900 mL) during the session.

Similar results were observed in both young and adult padel players. A recent literature review [4] on elite and amateur players of both sexes reported average heart rate (HR) values during matches ranging from 130 to 151 bpm (68–74% of maximum HR) for amateur players and from 154 to 179 bpm (80–85% of maximum HR) for elite players. Maximum heart rates during matches were recorded between 154 and 179 bpm, with approximately 97.75% of match time spent in the aerobic zone. In male professional players from the World Padel Tour, aged 26.3 ± 8.2 years, game simulations recorded an average HR of 145.4 ± 18.2 bpm, representing 72.7 ± 9.8% of their maximum HR obtained through an incremental test [2]. The significant body mass loss observed in our study subjects aligns with findings from a study on adult padel players, which documented an average weight loss of 700 g during match play [38]. Regarding the distances covered by our subjects during training, they covered 1.95 ± 0.37 km, with maximum speeds of 10.51 ± 1.25 km/h. Amateur players aged 35 ± 7 years covered 2.057 ± 0.327 km, reaching maximum speeds of 15.14 km/h [39]. These results suggest that in training match scenarios, master padel athletes can achieve workloads similar to those of younger and adult athletes, through challenging yet safe training, staying within healthy intensity zones. Our participants were trained individuals, and for older beginners, gradual progression is key to fostering aerobic adaptation [40], allowing them to develop both technical and physical skills. This makes padel practice a healthy and sustainable activity for older adults. Our study showed a trend suggesting that subjects who trained more hours per week tended to spend less time in elevated heart rate zones.

### 4.2. Profile of Mood States Evolution

Although the training indicated an impact in terms of fatigue, the overall mood state (POMS) of the subjects in this study was not affected by the exertion, and there was even an improvement in hostility levels, which decreased significantly (Wilcoxon test: Z = −2.032, *p* = 0.042). As this exertion was purely aerobic, these results are not surprising, since the effect of moderate acute exercise has already been associated with improvements in mood state (POMS) in young, adult [41,42], and even elderly persons [43]. For padel, we found only a master’s thesis where mood state levels were also not affected by training, but no improvement was noted [44]. Dehydration has been linked to mood deterioration, both in young and elderly populations. However, in older adults, pre-existing conditions such as depression or cognitive decline may amplify these effects [45,46]. In our study, POMS scores remained stable throughout the training session, suggesting no significant effect of dehydration on mood. Additionally, the lower total mood disturbance scores recorded both before and after training indicate that the participants were in a positive mood state, reducing the likelihood of any substantial influence from pre-existing conditions.

### 4.3. Hydration Evolution

The mean urine osmolality values recorded the night before, in the morning, pre-training, and post-training were 797.50, 778.33, 772.50, and 800.00 mOsm/kg, respectively. These values did not show any statistically significant differences and are indicative of either a normal hydration state [25,26] or minimal dehydration, as suggested by other authors [24,47]. On the other hand, urine color values were 2.7, 2.9, 2.8, and 3.8 AU, respectively, and significant statistical differences were obtained between post-training (indicating dehydration) compared to all other time points (indicating normal hydration). These results suggest that while the overall hydration levels of our subjects were adequate, dehydration may have increased during training.

Studies report that between 14% and 60% of elderly individuals do not meet daily hydration recommendations, which are 2 L for men and 1.6 L for women [48]. However, according to osmolality and color values, this does not appear to be the case for our subjects, except after training. One possible explanation is that our subjects were athletes, as research shows that more active older individuals tend to drink more than inactive ones, maintaining better hydration [49]. On the other hand, six of our subjects (50%) showed osmolality levels indicative of light to mild dehydration at least once, while two subjects (16.7%) had one or more measurements classified as dehydration, with one of them showing this only after training. These findings suggest that the hydration levels of our subjects were suboptimal, particularly the night before the study. In addition to focusing on hydration in the hours before and during training, these athletes should increase their fluid intake throughout the day and outside of training to meet the recommended daily liquid intake guidelines.

Our subjects consumed an average of 438 mL of liquids between dinner and bedtime, and 333 mL from waking up to breakfast, which may not have been sufficient to maintain hydration levels both upon waking and before training. However, while the correlations between fluid intake and urine osmolality were negative, they were not statistically significant. Nutritional guidelines for older athletes recommend consuming 400 to 600 mL of fluids 2 to 3 h before exercise [50], and the recommendation on exercise and fluid replacement suggests drinking 5–7 mL per kg of body mass [24] or more if the urine is dark [51] to ensure normohydration before training. With 333 mL consumed in the hours before training, which equates to approximately 4.071 mL per kg of body mass, it would have been safer for our subjects to consume slightly more fluids in the hours leading up to training.

During exercise, the recommended fluid intake is 400–800 mL per hour [19]. In our study, participants consumed 900 ± 283 mL of water over a 90 min session, averaging 600 mL per hour, which aligns with these guidelines. Studies of older individuals (aged 54–70 years) performing cycling at 65% VO_2_ peak in the heat, with ad libitum access to water, showed that participants consumed about 975 mL, maintaining their body mass throughout [52]. This aligns with our findings, suggesting that 600 mL per hour may be sufficient to maintain hydration in older populations during training. However, the significant increase in urine color post-training, indicating dehydration, suggests that higher fluid intake may be beneficial. There was a trend, though not statistically significant, that those who consumed more fluids experienced less dehydration and tended to train at higher intensities.

The increased risk of dehydration during exercise in older adults, compared to younger athletes, can be attributed to their reduced ability to dissipate heat due to lower sweat rates and reduced blood flow, along with diminished renal function, impaired detection of blood volume and plasma osmolality changes, higher baseline and greater increases in plasma osmolality, and a reduced thirst response, leading to insufficient fluid intake [53,54].

Although some dehydration was observed in our study, this highlights the need for practitioners to guide older adults, including athletes, in meeting their recommended fluid intake. For older adults, a daily intake of 1.5–2.0 L (6–8 glasses) is recommended, regardless of body weight. It is crucial to ensure that patients with conditions like chronic heart failure, especially those on diuretics, are not dehydrated. However, there is no evidence that consuming more than this amount provides additional health benefits; older adults should also be aware of the risk of acute water intoxication, which can occur if large volumes (0.7–1.0 L/h) are consumed quickly, exceeding the kidneys’ excretion capacity [48].

Strategies to increase fluid intake in older individuals include behavioral interventions, such as verbal prompts and increasing the availability, variety, and frequency of offered beverages, which have been shown to improve hydration [55]. To provide variety, ESPEN recommends offering fluids that have a hydrating effect, such as water, sparkling or flavored water, hot or cold tea, coffee, milk and milk-based drinks, fruit juices, soups, sports or soft drinks, smoothies, and alcoholic beverages with up to 4% alcohol. Social interaction and offering drinks based on personal preferences are key factors in encouraging fluid intake in older adults [56]. However, caution is advised for those with certain medical conditions, such as diabetes [57].

For older athletes, the recommended fluid intake is approximately 177 mL every 15 min [47], while the general athletic population is advised to consume 100–200 mL every 15 min [51,58]. In our study, senior padel players hydrated consistently, with water intake measured at 30, 60, and 90 min showing no statistically significant differences (Friedman test: Chi-square 1.85, *p* = 0.397). On average, they consumed approximately 151 mL every 15 min. This consistent hydration pattern demonstrates sensible behavior among our veteran athletes, who managed their fluid intake without affecting their sensation of satiety (SLIM scale), maintaining comfort throughout the training session. During the training session, the loss of body mass was 0.49%, which is well below the 2% dehydration threshold that should be avoided. This highlights that senior athletes may be following the current recommendations to maintain their hydration levels.

The correlations between urine osmolalities and urine color were statistically significant, with mostly large effects, indicating that urine color is an efficient, easy-to-use, and cost-effective alternative for monitoring hydration levels in our population of older padel players.

### 4.4. Limitations of the Study

Admittedly, our study has some limitations that should be acknowledged, such as the small number of subjects and the absence of female participants.

One of the study limitations is the fact that we did not measure participants’ stamina levels and playing history (including frequency of play), which could influence this study’s results and conclusions. Measuring participants’ stamina through an aerobic test prior to this study would be a valuable addition and could significantly influence this study’s outcomes.

For practical reasons, we were limited to using the simplest instruments to evaluate hydration status, foregoing more comprehensive methods like blood, 24 h urine collection, saliva, and bioimpedance analysis, which could have provided more detailed information.

It would have been beneficial to maintain nutritional diaries to gain a complete understanding of the diet and hydration of the subjects, especially on the day before training. However, to minimize the burden on the participants and to obtain accurate information, we decided to focus on hydration closer to the training session.

Although we provided recommendations on foods to avoid from the day before training until its start, incorporating a food diary to track potential influences on urine osmolality and color, as well as including total water consumption as a variable, would have added valuable insights to this study.

Studying the rehydration patterns of the subjects after training would have added depth to our findings, but for the same reasons, we chose not to include this information.

Additionally, we are always subject to the risk of over- or underestimating the descriptions of the participants.

### 4.5. Practical Implications and Suggestions for Future Studies

Even in mild conditions, most of our athletes tend to experience dehydration, primarily due to insufficient fluid intake before and after training. It is crucial for them to follow daily hydration recommendations outside of training sessions. As a practical insight from this study, to improve hydration in training and games, older male padel players should drink the recommended 2 L the day before. In the hours leading up to training, they should consume 5 to 7 mL of liquids per kilogram of body mass. During training, they should drink between 150 and 200 mL every 15 min. They should also monitor their hydration using a color scale and a sensation of satiety scale to adapt these recommendations to their individual needs. Trainers play a crucial role in promoting successful hydration by educating athletes both generally and during training. They should teach athletes how to use simple monitoring tools and assess their hydration habits. During training, trainers must also create opportunities that encourage regular and adequate hydration, ensuring athletes follow recommendations without exceeding them.

Further studies are needed to better understand the hydration needs of older athletes across different sports, analyzing them over various days and comparing different seasons and phases of their training plans. It would also be valuable to assess the impact of improved hydration in this population. In padel specifically, it would be interesting to examine hydration patterns across different age groups, genders, and levels of practice.

## 5. Conclusions

From our observation of a group of 12 senior male federated padel players, we obtained the first results regarding the characterization of hydration habits, physiological demands, and psychological responses before and during a padel training session. This contribution is valuable for developing tailored nutritional strategies, health monitoring, and performance optimization.

During a 90 min padel training session that included games, healthy older male participants exerted an average of 73.2 ± 12.3% of their maximum heart rate (107.00 ± 19.11 bpm), representing predominantly moderate aerobic effort. There were brief peaks at the anaerobic threshold (15% of the training time) and at maximum aerobic effort (5.5% of the training time), with heart rates averaging no more than 88.5 ± 11.7% of their maximum (130.00 ± 18.78 bpm) and an average heart recovery rate of 13.25 ± 8.7 bpm per minute. They achieved workloads similar to those of younger and adult athletes through challenging yet safe training, staying within healthy intensity zones while covering a reasonable distance of 1.95 ± 0.37 km and reaching maximum speeds of 10.51 ± 1.25 km/h. There was a trend for subjects who trained more hours per week tended to spend less time in elevated heart rate zones.

The mean urine osmolality values recorded at various times remained consistent, indicating normal hydration or minimal dehydration. However, urine color values showed significant differences, with the post-training value indicating dehydration at that moment. However, the hydration levels of some subjects were suboptimal, particularly during the night before the study.

Our subjects drank an average of 438 mL of liquids at night and 333 mL before breakfast, which may not have been sufficient to maintain hydration levels before training. With 333 mL consumed before training (around 4.071 mL per kg of body mass), the intake was slightly below the recommended guidelines, suggesting that higher consumption in the hours before training could have been safer for ensuring proper hydration.

During the training session, participants consumed 900 ± 283 mL over 90 min, averaging 600 mL per hour, which aligns with the guidelines of 400–800 mL per hour. These results suggest that 600 mL per hour may be sufficient for adequate hydration in senior padel athletes, but the significant increase in urine color indicates potential dehydration, suggesting that slightly higher fluid intake could be beneficial. Additionally, athletes who consumed more fluids tended to experience less dehydration and trained in higher intensity zones.

Senior padel players maintained consistent hydration throughout their training, averaging 151 mL every 15 min, with no statistically significant differences in water intake at 30, 60, and 90 min. This consistent pattern reflects sensible behaviors, as they managed their fluid intake without impacting their sensation of satiety. The average body mass loss during training was only 0.49%, well below the 2% dehydration threshold, suggesting that senior athletes effectively followed hydration recommendations of 100–200 mL every 15 min.

Medium fatigue subscale (POMS) values ranged from 3.42 ± 4.76 to 6.67 ± 4.62 between the start and end of the session, suggesting that the training, despite being of moderate intensity, had a significant impact on the players. The mood state of the older padel players remained stable throughout the training, with a decrease in hostility levels. However, we did not find any significant correlation between hydration levels and POMS results.

For older male padel players, it appears suitable to follow the American College of Sports Medicine (ACSM) hydration recommendations before and during training.

The significant correlations obtained with urine osmolality indicate that urine color is an effective, easy-to-use, and cost-effective method for monitoring hydration levels in this population.

## Figures and Tables

**Table 1:** Characterization of padel training effort.

Variables	Mean ± Standard Deviation
Average heart rate (bpm/%max. HR)	107.00 ± 19.11/73.17 ± 12.31
Maximum training heart rate (bpm %max. HR)	130.00 ± 18.78/88.50 ± 11.69
During training, 1 min HR recovery (bpm)	13.25 ± 8.70
Low effort zone (% of training time)	6.50 ± 10.84
Zone 1—very light effort (% of training time)	16.30 ± 23.90
Zone 2—light effort (% of training time)	29.20 ± 22.91
Zone 3—moderate effort (% of training time)	30.30 ± 26.73
Zone 4—high effort (% of training time)	15.00 ± 23.03
Zone 5—maximum effort (% of training time)	5.50 ± 11.49
Distance covered (km)	1.95 ± 0.37
Maximum speed reached (km/h)	10.51 ± 1.25

HR = heart rate, bpm = beats per minute, %max. HR = percentage of theoretical maximum heart rate of 220 − age.

**Table 2:** Evolution of urine osmolality, urine color, and body mass.

Variables	Mean ± Standard Deviation	Freidman (F) Wilcoxon (W)
Urine osmolality on the night before (mOsm·kg^−1^)	797.50 ± 213.42	(F) *p* = 0.776 (ns) Chi-square = 1.103
Urine osmolality in the morning upon waking (mOsm·kg^−1^)	778.33 ± 190.35	
Pre-training urine osmolality (mOsm·kg^−1^)	772.50 ± 178.64	
Post-training urine osmolality (mOsm·kg^−1^)	800.00 ± 207.32	
Urine color at night (AU)	2.70 ± 1.20	(F) *p* = 0.004 ** Chi-square = 13.286
Urine color in the morning (AU)	2.90 ± 1.60	
Urine color pre-training (AU)	2.80 ± 1.00	
Urine color post-training (AU)	3.80 ± 1.30	
Pre-training body mass (kg)	81.79 ± 7.44	(W) *p* = 0.016 **, Z = − 2.405
Post-training body mass (kg)	81.40 ± 7.25	

ns = not significant, AU = arbitrary units, ** highly significant *p* < 0.01.

**Table 3:** Subjects’ liquid intake at night, in the morning until the training, and during the training.

Variables	Mean ± Standard Deviation	Freidman (F)
Liquids consumed at night (liters)	0.43 ± 0.15	
Liquids consumed from waking up until breakfast (liters)	0.33 ± 0.27	
Water consumed up to 30 min of training (liters)	0.22 ± 0.18	(F) *p* = 0.394 ns Chi-square= 1.850
Water consumed between 30 and 60 min of training (liters)	0.35 ± 0.14	
Water consumed between 60 and 90 min of training (liters)	0.32 ± 0.19	
Total water consumed during training (liters)	0.90 ± 0.28	

ns = not significant.

**Table 4:** Subjects’ satiety level results before and after the training.

Variables	Mean ± Standard Deviation	Wilcoxon (W)
Pre-training satiety (AU)	6.67 ± 19.70	(W) *p* = 0.136 ns, Z = −1.490
Post-training satiety (AU)	−8.33 ± 18.01	

AU = arbitrary units, ns = not significant.

**Table 5:** Evolution of mood profile (POMS) during the training.

Variables (AU)	Mean ± Standard Deviation	Wilcoxon (W)
Pre-Training Tension	4.83 ± 2.89	*p* = 0.108 ns Z = −1.605
Post-Training Tension	3.08 ± 1.98	
Pre-Training Depression	0.67 ± 1.07	*p* = 0.109 ns Z = −1.604
Post-Training Depression	0.17 ± 0.39	
Pre-Training Hostility	2.00 ± 2.37	*p* = 0.042 * Z = −2.032
Post-Training Hostility	0.83 ± 1.19	
Pre-Training Fatigue	3.42 ± 4.76	*p* = 0.024 * Z = −2.256
Post-Training Fatigue	6.67 ± 4.62	
Pre-Training Confusion	5.08 ± 3.18	*p* = 0.964 ns Z = −0.045
Post-Training Confusion	5.33 ± 2.49	
Pre-Training Vigor	16.17 ± 6.44	*p* = 1.000 ns Z = 0.000
Post-Training Vigor	16.08 ± 6.33	
Pre-Training Total Mood Disturbance	32.83 ± 12.96	*p* = 0.906 ns Z = −0.118
Post-Training Total Mood Disturbance	32.75 ± 11.05	

ns = not significant, AU = arbitrary units, * significant *p* < 0.05.

## Data Availability

The data presented in this study are available on request from the corresponding author, due to privacy or ethical restrictions.
